# ERICA: patterns of alcohol consumption in Brazilian adolescents

**DOI:** 10.1590/S01518-8787.2016050006684

**Published:** 2016-02-02

**Authors:** Evandro Silva Freire Coutinho, Debora França-Santos, Erika da Silva Magliano, Katia Vergetti Bloch, Laura Augusta Barufaldi, Cristiane de Freitas Cunha, Maurício Teixeira Leite de Vasconcellos, Moyses Szklo

**Affiliations:** I Departamento de Epidemiologia e Métodos Quantitativos em Saúde. Escola Nacional de Saúde Pública Sérgio Arouca. Fundação Oswaldo Cruz. Rio de Janeiro, RJ, Brasil; II Instituto de Medicina Social. Universidade do Estado do Rio de Janeiro. Rio de Janeiro, RJ, Brasil; III Programa de Pós-Graduação em Saúde Coletiva. Universidade Federal do Rio de Janeiro. Rio de Janeiro, RJ, Brasil; IV Departamento de Vigilância de Doenças e Agravos Não Transmissíveis e Promoção da Saúde. Secretaria de Vigilância em Saúde. Ministério da Saúde. Brasília, DF, Brasil; V Hospital de Clínicas. Universidade Federal de Minas Gerais. Belo Horizonte, MG, Brasil; VI Escola Nacional de Ciências Estatísticas. Fundação Instituto Brasileiro de Geografia e Estatística. Rio de Janeiro, RJ, Brasil

**Keywords:** Adolescent, Alcohol Drinking epidemiology, Prevalence, Cross-Sectional Studies

## Abstract

**OBJECTIVE:**

To describe the patterns of alcohol consumption in Brazilian adolescents.

**METHODS:**

We investigated adolescents who participated in the Study of Cardiovascular Risks in Adolescents (ERICA). This is a cross-sectional, national and school-based study, which surveyed adolescents of 1,247 schools from 124 Brazilian municipalities. Participants answered a self-administered questionnaire with a section on alcoholic beverages consumption. Measures of relative frequency (prevalence), and their 95% confidence intervals, were estimated for the following variables: use of alcohol beverages in the last 30 days, frequency of use, number of glasses or doses consumed in the period, age of the first use of alcohol, and most consumed type of drink. Data were estimated for country and macro-region, sex, and age group. The module survey of the Stata program was used for data analysis of complex sample.

**RESULTS:**

We evaluated 74,589 adolescents, who accounted for 72.9% of eligible students. About 1/5 of adolescents consumed alcohol at least once in the last 30 days and about 2/3 in one or two occasions during this period. Among the adolescents who consumed alcoholic beverages, 24.1% drank it for the first time before being 12 years old, and the most common type of alcoholic beverages consumed by them were drinks based on vodka, rum or tequila, and beer.

**CONCLUSIONS:**

There is a high prevalence of alcohol consumption among adolescents, as well as their early onset of alcohol use. We also identified a possible change in the preferred type of alcoholic beverages compared with previous research.

## INTRODUCTION

Alcohol is the most widely used psychotropic substance among adolescents from Brazil and around the world^6^. The consumption of alcoholic beverages in this group is troubling, both for their greater tendency towards impulsive behaviors in this phase of life, and for damages to brain development caused by alcohol in childhood and adolescence. Above all, it compromises the cortical region, affecting negatively the cognitive, emotional and social developments of individuals^5^. The use of alcohol in adolescence tends to occur along with other risk behaviors regarding health, such as use of tobacco and illicit drugs, as well as potentially dangerous sexual practices^5^. A longitudinal study with Finnish adolescents observed use of alcohol increased the risk of smoking in adulthood^11^. In addition, the early onset of alcohol use is associated with future problems related to alcohol abuse^9^.

The Brazilian law forbids sale of alcoholic beverages to minors (people younger than 18 years of age). Since March 2015, those who sell, provide, serve, administer or deliver alcoholic beverages to children or adolescents (even when for free) are liable to imprisonment from two to four years and to payment of a fine^a^. Advertising is restricted to beverages with alcohol content equal or greater than 0.5 Gay Lussac degree (GL), whose advertisement can only be aired on radio and television between 9 p.m. and 6 a.m. Until 11 p.m., airing can only be made during breaks of programs not recommended for minors. Even so, studies have shown that a major portion of adolescents in Brazil consume alcoholic beverages^10^ ^,^ ^12^ ^,^ ^13^.

Studies on the use of alcohol in Brazil, having students as samples, began in the mid-1980s. Systematic review^1^ of 28 population studies with adolescents between 10 and 19 years found prevalences of alcohol consumption (according to different definitions) ranging from 23.0% to 68.0%.

The aim of this paper is to describe the patterns of alcohol consumption in Brazilian adolescents.

## METHODS

The Study of Cardiovascular Risks in Adolescents (ERICA) is a cross-sectional study that assessed adolescents aged between 12 and 17 years, enrolled in public and private schools of 273 Brazilian municipalities with more than 100,000 inhabitants. Data were collected between March 2013 and December 2014^2^.

In the sampling process, we stratified the population into 32 geographical strata, comprising the capitals of the 27 Federation units, and five strata comprising the municipalities of each one of the five macro-regions of the country. After geographical stratification, the sampling procedure had two stages: selection of the participating schools and selection of classes. The schools were selected in each geographical stratum with proportional probability to size and inversely proportional to the distance from the capital. In the second stage, three classes were selected from each school with equal probabilities during the fieldwork. Using the year of the class as age variable, only the seventh, eighth and ninth grades of elementary school and first, second and third grades of high school were eligible for the selection^14^.

We obtained the information by a questionnaire self-administered in the classroom, using an electronic data collector LG model GM750Q, under supervision of the study team. In addition to the sociodemographic variables, data concerning the consumption of alcoholic beverages were also used. These variables were measured by the following questions:

- Age at which you took at least a glass (or a dose) of alcohol for the first time.
- Days of ingestion of at least one glass (or a dose) of alcohol within the past 30 days.
- Glass or doses consumed, on average, in the last 30 days.
- Type of the most often consumed alcoholic beverage.

Measures of relative frequency (prevalence) and their respective 95% confidence intervals were calculated for alcohol use within the past 30 days, for the frequency of such use, the amount of glasses or doses consumed in the period, age at which alcohol was taken for the first time, and type of the most consumed beverage. All these data were estimated for the country and for macro-region, sex, and age group.

Prevalence estimates and confidence intervals were fixed considering the sampling design. Analyses were performed using the command svy (survey) for the complex data analysis of the Stata program 14.0^b^.

ERICA was conducted according to the principles of the Declaration of Helsinki. The study was approved by the Ethics Committees of the Universidade Federal do Rio de Janeiro (Process 45/2008) and of each one of the 26 states and the Federal District. We obtained the consents to carry out the study from the secretariats of education and schools. All students signed an assent form. When the Ethics Committee demanded, the informed consent of the children’s legal guardians even for students who would not undergo blood collection, was also sent for signing. The privacy and information confidentiality of students was guaranteed.

## RESULTS

In total, 74,589 adolescents answered the questionnaire. Of these, 55.3% were female. The average age was 14.7 years (SD = 1.6), and 78.7% studied in public schools. Table 1 presents the distribution by sex and age of samples and the estimated population after expansion of the sample^14^.

**Table 1:** Adolescents of ERICA sample and of the estimated population, according to IBGE by sex, age group, and macro-regions in municipalities with more than 100,000 inhabitants. ERICA, Brazil, 2013-2014.

Sex and age	Brazil	%	North	%	Northeast	%	Midwest	%	Southeast	%	South	%
ERICA sample												
Male	33,364	44.7	6,861	45.5	10,310	44.5	4,097	42.1	7,620	44.6	4,476	46.9
12-14 years	15,433	20.7	3,167	21.0	4,658	20.1	1,965	20.2	3,587	21.0	2,056	21.6
15-17 years	17,931	24.0	3,694	24.5	5,652	24.4	2,132	21.9	4,033	23.6	2,420	25.4
Female	41,225	55.3	8,212	54.5	12,857	55.5	5,630	57.9	9,460	55.4	5,066	53.1
12-14 years	18,708	25.1	3,739	24.8	5,853	25.3	2,547	26.2	4,322	25.3	2,247	23.6
15-17 years	22,517	30.2	4,473	29.7	7,004	30.2	3,083	31.7	5,138	30.1	2,819	29.5

Total	74,589	100	15,073	20.2	23,167	31.1	9,727	13.0	17.080	22.9	9,542	12.8

Estimated population												
Male	5,095,563	50.2	427,365	4.2	1,082,182	10.7	389,167	3.8	2,595,521	25.6	601,328	5.9
12-14 years	2,697,440	26.6	224,876	2.2	569,111	5.6	204,588	2.0	1,375,262	13.6	323,603	3.2
15-17 years	2,398,123	23.6	202,489	2.0	513,071	5.1	184,579	1.8	1,220,259	12.0	277,725	2.7
Female	5,052,137	49.8	427,997	4.2	1,082,851	10.7	388,843	3.8	2,557,985	25.2	594,461	5.9
12-14 years	2,650,761	26.1	225,587	2.2	562,205	5.5	202,923	2.0	1,344,038	13.2	316,008	3.1
15-17 years	2,401,376	23.7	202,410	2.0	520,646	5.1	185,920	1.8	1,213,947	12.0	278,453	2.7

Total	10,147,700	100.0	855,362	8.4	2,165,033	21.3	778,010	7.7	5,153,506	50.8	1,195,789	11.8

Population estimate source: http://www.ibge.gov.br/home/estatistica/populacao/projecao_da_populacao/2013/default.shtm

ERICA: Study of Cardiovascular Risks in Adolescents

Twenty-one percent of the adolescents consumed alcohol at least once in the last 30 days, the highest prevalence being observed in the South region (27.5%), and the smallest in the North region (14.8%) of Brazil. Prevalences were higher for adolescents aged between 15 and 17 years (Table 2).

**Table 2:** Prevalences and confidence intervals of alcoholic beverages consumption by macro-region, sex and age group. ERICA, 2013-2014.

Sex and age	Brazil		North		Northeast		Midwest		Southeast		South	
					
	%	95%CI	%	95%CI	%	95%CI	%	95%CI	%	95%CI	%	95%CI
Male	21.0	19.5-22.5	14.7	13.5-16.1	16.8	14.8-19.1	23.2	20.7-26.0	22.1	19.6-24.9	26.3	23.2-29.6
12-14 years	13.1	11.6-14.8	7.7	6.5-9.1	10.3	8.2-12.9	12.9	10.7-15.6	13.7	11.1-16.9	19.2	16.3-22.6
15-17 years	29.8	27.7-32.1	22.6	20.5-24.8	24.1	21.6-26.8	34.7	30.2-39.4	31.7	27.9-35.6	34.4	28.9-40.4
Female	21.5	20.3-22.7	14.9	13.7-16.2	16.5	15.1-18.1	23.3	21.5-25.1	22.7	20.6-24.9	28.8	25.8-32.1
12-14 years	14.4	13.1-15.8	10.8	9.4-12.2	10.8	9.3-12.2	14.8	13.3-16.4	15.3	13.1-17.8	19.3	16.1-23.1
15-17 years	29.3	27.3-31.4	19.5	17.8-21.5	22.8	20.6-25.1	32.5	29.2-36.0	30.9	27.2-34.9	39.6	35.7-43.7

Total	21.2	20.2-22.3	14.8	13.8-15.9	16.7	15.2-18.3	23.3	21.4-25.2	22.4	20.7-24.3	27.5	25.3-29.9

**Figure 1:**
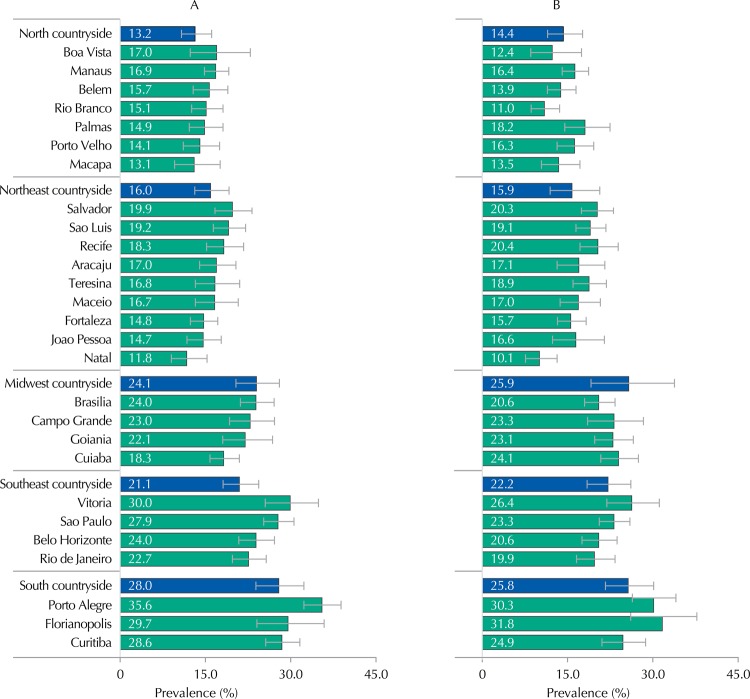
Prevalence and confidence intervals of alcohol consumption in female (A) and male (B) adolescents, by capital. ERICA, Brazil, 2013-2014.

These prevalences were similar when stratified by sex in the five macro-regions (Table 2). When data were stratified by capital, Porto Alegre, Florianopolis, and Vitoria presented the highest prevalence of alcohol use for both sexes. Natal was the capital with the lowest prevalence (Figure 1). In the case of countryside municipalities, the South and Midwest regions showed the highest prevalences for both sexes, while North and Northeast showed the lowest rates (Figure 1).

Among adolescents who have reported drinking alcohol in the last 30 days, approximately 68.0% have done so in one or two occasions in the period (Table 3). When comparing the consumption of once to twice a month with 10 or more times, male adolescents and those who are older used alcohol more frequently. The Midwest region was the exception, the proportion of male alcohol users being greater in the group that made use of it once or twice, and in the group of females, which showed strata from six to nine times in the last month.

**Table 3:** Frequency (number of days) of use of alcohol among adolescents who consumed alcohol in the past 30 days, by macro-region, sex, and age group. ERICA, Brazil, 2013-2014.

Frequency	Brazil		North		Northeast		Midwest		Southeast		South	
					
	%	95%CI	%	95%CI	%	95%CI	%	95%CI	%	95%CI	%	95%CI
1-2 days												
Male	67.0	64.3-69.5	71.2	66.8-75.3	68.5	63.4-73.2	68.2	63.3-72.8	65.8	61.1-70.1	65.5	60.2-70.4
12-14	73.0	68.7-76.9	78.6	71.4-84.4	74.0	64.4-81.7	78.7	72.3-84.0	71.2	64.1-77.4	71.4	61.4-79.7
15-17	60.2	57.3-63.0	62.9	58.6-67.1	62.4	58.3-66.3	56.6	50.0-63.0	59.7	54.5-64.7	58.6	54.3-62.7
Female	69.0	66.2-71.6	76.7	79.3 79.3-	73.1	69.4-76.5	61.5	56.6-66.3	67.2	62.0-72.0	68.0	64.5-71.4
12-14	74.1	69.6-78.2	83.5	79.2-87.1	79.1	73.5-83.7	62.2	53.0-70.6	71.2	62.5-78.5	78.7	73.8-82.9
15-17	63.2	60.4-66.0	69.1	64.3-73.5	66.7	62.8-70.5	60.8	56.0-65.4	62.8	57.7-67.7	55.9	50.4-61.3

Total	67.9	65.8-70.0	74.0	71.4-76.3	70.8	67.6-73.8	64.9	60.9-68.7	66.5	62.7-70.1	66.8	63.1-70.3

3-5 days												
Male	16.1	14.5-17.8	12.5	10.1-15.3	11.9	10.2-13.8	14.6	11.2-18.7	18.5	21.5-15.8	17.0	21.5-15.8
12-14	12.3	10.0-15.1	8.3	5.0-13.3	6.2	4.6-8.3	6.9	3.6-12.6	16.2	12.1-21.3	13.1	7.4-22.0
15-17	19.6	17.8-21.5	17.2	14.0-21.0	18.2	15.1-21.6	23.1	17.7-29.6	21.1	17.5-25.2	21.5	18.1-25.4
Female	16.6	14.8-18.5	12.3	10.2-14.7	14.5	11.8-17.7	16.3	13.1-20.0	17.8	14.7-21.4	18.1	15.5-21.1
12-14	13.8	11.0-17.3	8.8	6.1-12.6	12.6	8.5-18.4	14.3	9.9-20.2	15.5	10.6-22.1	12.1	8.6-16.7
15-17	19.6	17.8-21.6	16.1	13.2-19.5	16.5	13.6-19.9	18.5	14.7-22.9	20.4	17.3-23.8	25.0	21.0-29.6

Total	16.3	15.0-17.8	12.4	10.7-14.3	13.2	11.6-15.0	15.4	12.7-18.7	18.2	15.8-20.8	17.6	15.4-20.0

6-9 days												
Male	8.4	7.1-9.9	8.4	6.4-10.9	9.1	6.9-11.9	7.1	5.3-9.6	8.0	6.0-10.6	9.6	6.3-14.3
12-14	7.4	5.5-9.9	6.7	4.1-10.7	10.0	6.3-15.5	7.0	4.2-11.4	6.1	3.5-10.5	9.1	4.1-19.0
15-17	9.5	8.0-11.1	10.3	7.6-13.8	8.1	6.2-10.6	7.3	5.4-9.9	10.1	7.6-13.2	10.2	7.7-13.4
Female	7.7	6.5-9.1	5.4	3.7-7.7	7.6	5.7-10.0	10.5	7.6-14.4	7.6	5.6-10.3	8.0	6.0-10.6
12-14	5.9	4.3-8.0	3.5	1.7-7.1	3.8	8.0-1.8	11.4	6.6-18.8	6.4	3.9-10.5	5.5	3.2-9.2
15-17	9.6	8.2-11.3	7.4	4.3-12.4	11.6	9.3-14.4	9.5	6.7-13.5	8.9	6.6-12.0	10.8	8.3-14.0

Total	8.0	7.1-9.1	6.9	5.6-8.4	8.3	6.9-10.1	8.8	7.1-11.0	7.8	6.2-9.7	8.8	6.3-12.1

10 or + days												
Male	8.6	7.3-10.0	7.9	5.3-11.6	10.5	7.8-14.0	10.0	7.5-13.3	7.8	5.8-10.3	8.0	5.8-10.8
12-14	7.3	5.4-9.7	6.4	3.1-12.6	9.8	5.8-16.1	7.5	4.4-12.3	6.5	3.8-10.9	6.4	3.3-12.2
15-17	10.0	8.5-11.8	9.6	7.2-12.7	11.4	7.7-16.5	12.9	9.9-16.7	9.2	11.9-7.0	9.7	6.9-13.5
Female	6.8	5.8-8.0	5.7	4.4-7.2	4.8	3.5-6.5	11.7	8.4-16.0	7.4	9.6-5.6	5.8	4.4-7.7
12-14	6.1	4.6-8.1	4.1	2.4-6.9	4.4	2.5-7.9	12.1	7.1-20.1	6.9	4.4-10.5	3.7	1.9-7.0
15-17	7.5	6.3-9.0	7.4	5.7-9.5	5.1	3.9-6.7	11.2	8.3-14.9	7.9	5.7-10.9	8.2	6.1-11.0

Total	7.7	6.8-8.6	6.8	5.2-8.7	7.6	6.2-9.4	10.9	8.4-14.0	7.6	6.2-9.2	6.9	5.6-8.5

Among adolescents who consumed alcoholic beverages, 24.1% drank for the first time before the age of 12, being 26.6% (95%CI 22.3-25.9) male and 21.7% (95%CI 24.2-19.4) female. This pattern repeated itself in all of the five macro-regions of the country.

**Figure 2:**
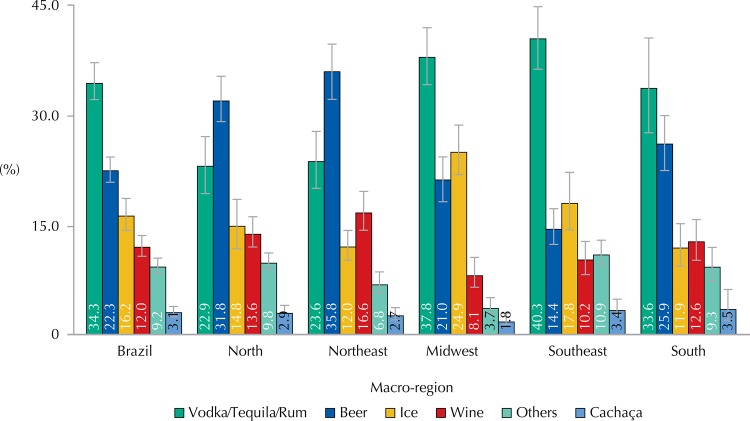
Types of alcoholic beverages consumed by adolescents by macro-region. ERICA, Brazil, 2013-2014.

The types of alcoholic beverages most often consumed by adolescents in the country were drinks based on vodka, rum or tequila, followed by beer (Figure 2). However, this pattern varied according to the macro-region. In the North and Northeast, beer was the most reported drink, while drinks based on vodka, rum or tequila were the most referred to in the Midwest, Southeast, and South regions. In general, we observed a higher proportion of male adolescents among the consumers of beer, and of female adolescents among consumers of Ice beverages (Table 4). The consumption of beer and drinks based on vodka, rum or tequila increased with age, while Ice beverages and wine decreased.

**Table 4:** Percentage of most consumed types of beverages by adolescents in the past 30 days, by sex and age. ERICA, Brazil, 2013-2014.

Variable	Beer		Wine		Ice		Cachaça		Vodka, tequila and rum		Others	
					
	%	95%CI	%	95%CI	%	95%CI	%	95%CI	%	95%CI	%	95%CI
Male	25.1	22.4-27.9	11.1	9.5-13.0	12.4	10.9-14.2	3.6	2.7-4.7	35.3	32.3-38.5	8.4	7.1-10.0
12-14	23.1	19.3-27.4	13.6	11.0-16.7	13.3	11.4-15.5	3.4	5.5-2.1	30.1	26.3-34.3	10.9	8.5-13.8
15-17	27.3	24.8-29.9	8.3	7.0-10.0	11.5	9.2-14.2	3.7	2.5-5.4	41.1	37.6-44.8	5.7	4.6-6.9
Female	19.5	17.7-21.5	12.9	11.0-15.0	19.9	16.9-23.4	2.7	2.0-3.6	33.3	30.4-36.4	10.0	8.5-11.7
12-14	15.5	13.5-17.8	14.4	11.4-18.1	22.8	18.4-27.8	2.2	1.3-3.7	29.5	25.7-33.7	13.4	10.9-16.3
15-17	24.0	21.2-27.0	11.2	9.6-12.9	16.8	13.8-20.3	3.2	2.3-4.5	37.5	33.6-41.5	6.3	5.0-7.8

## DISCUSSION

The prevalence of alcoholic beverages consumption in at least one occasion in the last 30 days, found in ERICA (21.0%), was lower than the one observed in Brazilian students in the National Survey of School Health (PeNSE)*,* held in 2009 (27.3%)^10^ and 2012 (26.1%)^c^. However, the sample of PeNSE was restricted to ninth-grade students and approximately 90.0% of them were aged between 13 and 15 years.

We observed no difference between the data of prevalence of ERICA and those found in the sixth survey with elementary and high school students of 27 Brazilian state capitals, held in 2010^3^.

Pinsky et al.^12^ (2010) reported a prevalence of 24.2% in a sub-sample of 661 Brazilian adolescents aged between 14 and 17 years, who were part of the *I Levantamento Nacional de Álcool e Drogas* (LENAD – First National Alcohol and Drugs Survey), held in 2005-2006.

Prevalences of use of alcohol found in Brazil are lower than those estimated in a national study conducted in the United States in 2013 – Youth Risk Behavior Survey, in which about 35.0% of adolescents between 14 and 17 years old reported drinking alcohol in the last 30 days^7^. Data from the study Health Behavior in School-Aged Children (HBSC) for adolescents with 11 to 15 years from 41 countries and regions of Europe and North America are more difficult to be compared with ours, since they are presented as weekly consumption, ranging from 0% to 59.0%, depending on the country, sex and age group^5^.

As well as in our study, data on adolescents from LENAD 2005-2006 and PeNSE 2012 showed higher prevalence of alcohol use in adolescents residing in Southern Brazil, and lower for those in the North region. However, in the case of LENAD 2005-2006, the authors combined the North and Midwest macro-regions in the same stratum^12^. A study with adolescents aged between 11 and 15 years from the general population of the city of Pelotas, in Southern Brazil, found prevalence of alcohol use of 23.0% in the past month^13^. The prevalence of alcohol use in this study, as well as in those reported by Pinsky et al.^12^, in LENAD 2005-2006, and by Malta et al.^10^, in PeNSE, increased with age, which suggests consumption maintenance by those who started early and accession of new users over time.

Our results, when estimated by macro-region, are consistent with the findings of Pinsky et al.^12^ (2010) and Strauch et al.^13^ (2009), which did not observe differences in prevalence of alcohol users in the past 30 days among male and female adolescents. However, male adolescents seem to make a more intense use of it, especially when taking into account the monthly frequency of 10 times or more.

A quarter of the adolescents who reported alcohol consumption in the last month in this study, took it for the first time before the age of 12, this proportion being greater among male adolescents. Other national and international studies also reported an early onset in the experimentation of alcoholic beverages^3^ ^,^ ^7^ ^,^ ^10^ ^,^ ^12^. This is disturbing when considering that the consumption of alcoholic beverages is only legal in Brazil for individuals aged 18 years or more, and that early initiations have been linked with increased risks of unintentional injuries, car accidents, sexual risk behaviors, use of tobacco and illicit drugs, as well as cognitive impairment in adult life^9^ ^,^ ^11^.

When we analyze the types of most commonly consumed beverages, it seems that the preference has been changing. Our data were very different from those reported by Pinsky et al.^12^, in LENAD 2005-2006 adolescents sub-sample. In the latter, the beverage reported as the most consumed was beer (about 50.0%), followed by wine (about 35.0%). Overall, our study found a greater consumption of distillates (vodka, rum, and tequila), especially in the Midwest, Southeast, and South regions. Even in the North and Northeast, where beer was the most consumed beverage, drinks based on vodka, rum or tequila came second, exceeding 20.0% in the proportion of most consumed drinks in the last month, regardless of sex. Considering the South is a region of cooler climate and also a wine producer, wine were the third in the rank of most consumed beverages, with about 13.0%. Therefore, we observed likely changes in the favorite type of beverage of Brazilian adolescents, who have been opting for high alcohol content drinks. Such hypothesis deserves to be investigated in future studies with this population group, along with the motivations for such behavior. An explanation we should consider is that adolescents have been searching for more tasty alcohol beverages, which is easily obtained by mixing it with soft drinks, juices, and milky and. A study with 350 young people from Sydney, Australia, identified these drinks as the favorite ones among this age group^4^. Another aspect we should highlight regarding the consumption of this type of beverage is that, since it contains a higher alcohol concentration (about 40.0%), distilled beverages provide the effects of high alcohol intakes even with the consumption of small amounts.

ERICA data represent the consumption profile of adolescents attending schools and residing in medium or large municipalities, i.e., with more than 100,000 inhabitants. Nevertheless, these findings are close to those concerning adolescents of the general population of Brazil, since the access to school is of about 95.0% of the population from six to 14 years old, and of 85.0% from 15 to 19 years old^d^.

Concluding, we observed high prevalence of use of a substance whose consumption is prohibited by law in the age group covered by this study. Additionally, the data showed early onset of alcohol use and possible changes in preferences compared to previous research. However, the results for studies with student populations should be considered carefully when the aim is to generalize the findings to populations that may be outside the school environment. For this reason, studies with the general population of adolescents are needed to better investigate such trends.

The understanding of consumption patterns is essential to eliminate preconceived ideas when it comes to the most affected groups and the usage patterns of alcohol by adolescents, providing subsidies for the development of public policies of prevention and treatment. According to Laranjeira et al.^8^, most of the Brazilian adult population supports the increase of preventive and restrictive programs to the use of alcohol to be carried out in schools and government campaigns. Given that, there is a need for policies accompanied by continuous effectiveness assessments to regulate the alcohol market and avoid the early consumption of alcoholic beverages.

## Footnotes

a Brasil. Lei nº 13.106, de 17 de março de 2015. Altera a Lei nº 8.069, de 13 de julho de 1990 - Estatuto da Criança e do Adolescente, para tornar crime vender, fornecer, servir, ministrar ou entregar bebida alcoólica a criança ou a adolescente; e revoga o inciso I do art. 63 do Decreto-Lei nº 3.688, de 3 de outubro de 1941 - Lei das Contravenções Penais. Brasília (DF); 2015 [cited 2015 Nov 12]. Available from: http://www.planalto.gov.br/ccivil_03/_Ato2015-2018/2015/Lei/L13106.htm

b Stata Corp. Stata: statistical software for professionals. Version 14.0. College Station, Texas; 2015.

c Instituto Brasileiro de Geografia e Estatística. Pesquisa Nacional de Saúde do Escolar: 2012. Rio de Janeiro (RJ): IBGE; 2013.

d Instituto Brasileiro de Geografia e Estatística. Pesquisa Nacional de Saúde do Escolar: 2012. Rio de Janeiro (RJ): IBGE; 2013.
