# Supplementary material for: ERICA: patterns of alcohol consumption in Brazilian adolescents
Source: Rev Saude Publica. 2016 Feb 2;50(Suppl 1):8s. doi: 10.1590/S01518-8787.2016050006684 (PMC4767031; doi:10.1590/S01518-8787.2016050006684)
Supplement: Translation

# Translation: ERICA: padrões de consumo de bebidas alcoólicas em adolescentes brasileiros

**Authors:** Evandro Silva Freire Coutinho, Debora França-Santos, Erika da Silva Magliano, Katia Vergetti Bloch, Laura Augusta Barufaldi, Cristiane de Freitas Cunha, Maurício Teixeira Leite de Vasconcellos, Moyses Szklo

**Affiliations:** I Departamento de Epidemiologia e Métodos Quantitativos em Saúde. Escola Nacional de Saúde Pública Sérgio Arouca. Fundação Oswaldo Cruz. Rio de Janeiro, RJ, Brasil; II Instituto de Medicina Social. Universidade do Estado do Rio de Janeiro. Rio de Janeiro, RJ, Brasil; III Programa de Pós-Graduação em Saúde Coletiva. Universidade Federal do Rio de Janeiro. Rio de Janeiro, RJ, Brasil; IV Departamento de Vigilância de Doenças e Agravos Não Transmissíveis e Promoção da Saúde. Secretaria de Vigilância em Saúde. Ministério da Saúde. Brasília, DF, Brasil; V Hospital de Clínicas. Universidade Federal de Minas Gerais. Belo Horizonte, MG, Brasil; VI Escola Nacional de Ciências Estatísticas. Fundação Instituto Brasileiro de Geografia e Estatística. Rio de Janeiro, RJ, Brasil

**Keywords:** Adolescente, Consumo de Bebidas Alcoólicas, epidemiologia, Prevalência, Estudo Transversal

## Abstract

**OBJETIVO:**

Descrever padrões de consumo de bebidas alcoólicas em adolescentes brasileiros.

**MÉTODOS:**

Foram investigados adolescentes participantes do Estudo de Riscos Cardiovasculares em Adolescentes (ERICA). Trata-se de estudo transversal, multicêntrico, nacional e de base escolar, que avaliou adolescentes de 1.247 escolas em 124 municípios brasileiros. Os participantes responderam questionário autoaplicável que incluía seção sobre consumo de bebidas alcoólicas. Foram calculadas medidas de frequência relativa (prevalências) e respectivos intervalos de confiança de 95% para as seguintes variáveis: uso de bebidas alcoólicas nos últimos 30 dias, frequência de uso, número de copos ou doses consumidas no período, idade com que bebeu pela primeira vez e tipo de bebida mais consumido. Os dados foram estimados para o País e por macrorregião, sexo e grupo etário. Utilizou-se o módulo *survey* do programa Stata para análise de dados de amostra complexa.

**RESULTADOS:**

Foram avaliados 74.589 adolescentes, que representaram 72,9% dos alunos elegíveis. Cerca de 1/5 dos adolescentes consumiram bebidas alcoólicas pelo menos uma vez nos últimos 30 dias e, desses, aproximadamente 2/3 o fizeram em uma ou duas ocasiões no período. Entre os adolescentes que consumiam bebidas alcoólicas, 24,1% beberam pela primeira vez antes de 12 anos de idade, e os tipos de bebidas alcoólicas mais consumidas pelos adolescentes foram os drinques à base de vodca, rum ou tequila e a cerveja.

**CONCLUSÕES:**

Observou-se prevalência elevada de uso de álcool por adolescentes, assim como um início precoce. Identificou-se ainda uma possível mudança do tipo de bebida de preferência em comparação com investigações anteriores.

## INTRODUÇÃO

O álcool é a substância psicotrópica mais utilizada por adolescentes no Brasil e no mundo^6^. O consumo de bebidas alcoólicas nesse grupo é preocupante, tanto pela maior tendência à impulsividade nessa fase da vida, quanto pelo prejuízo ao desenvolvimento cerebral na infância e na adolescência causado pelo álcool. Este compromete sobretudo a região cortical, afetando negativamente o desenvolvimento cognitivo, emocional e social do indivíduo^5^. O uso de álcool na adolescência tende a ocorrer em conjunto com outros comportamentos de risco para a saúde, como o uso de tabaco e de drogas ilícitas, além de comportamento de risco sexual^5^. Estudo longitudinal com adolescentes finlandeses observou que o uso de álcool na adolescência aumentava o risco de tabagismo na vida adulta^11^. Além disso, o início precoce do uso de álcool está associado com problemas futuros relacionados ao uso abusivo de álcool^9^.

A legislação brasileira proíbe a venda de bebidas alcoólicas a menores de 18 anos. Desde março de 2015, vender, fornecer, servir, ministrar ou entregar bebida alcoólica a criança ou adolescente, ainda que gratuitamente, é passível de detenção por dois a quatro anos e multa^a^. A publicidade está restrita a bebidas com teor alcoólico igual ou superior a 0,5 grau Gay Lussac (GL), cujos anúncios só podem ser veiculados em emissoras de rádio e televisão entre 21h e 6h. A veiculação até 23h só pode ser feita no intervalo de programas não recomendados para menores de 18 anos. Mesmo assim, estudos têm mostrado o consumo de bebidas alcoólicas por uma parcela importante de adolescentes no País^10^ ^,^ ^12^ ^,^ ^13^.

Os estudos sobre o uso de álcool no Brasil, com amostras de estudantes, começaram em meados da década de 1980. Revisão sistemática^1^ de 28 estudos populacionais com adolescentes entre 10 e 19 anos encontrou prevalências de consumo de bebidas alcoólicas (segundo diferentes definições) variando de 23,0% a 68,0%.

O objetivo deste artigo é descrever os padrões de consumo de bebidas alcoólicas em adolescentes brasileiros.

## MÉTODOS

O Estudo de Riscos Cardiovasculares em Adolescentes (ERICA) é um estudo transversal que avaliou adolescentes com idades entre 12 e 17 anos, matriculados em escolas públicas e privadas de 273 municípios brasileiros com mais de 100.000 habitantes. Os dados foram coletados entre março de 2013 e dezembro de 2014^2^.

No processo amostral, a população foi estratificada em 32 estratos geográficos, constituídos pelas capitais das 27 unidades da Federação e cinco estratos que compreenderam municípios de cada uma das cinco macrorregiões do País. Após a estratificação geográfica, o processo de amostragem foi conduzido em duas etapas: seleção das escolas e seleção das turmas. As escolas foram selecionadas em cada estrato geográfico com probabilidade proporcional ao tamanho, e inversamente proporcional à distância da capital. Na segunda etapa, três turmas em cada escola sorteada foram selecionadas com probabilidades iguais durante o trabalho de campo. Usando-se o ano da turma como variável informativa da idade, apenas as classes dos sétimo, oitavo e nono anos do ensino fundamental e primeiro, segundo e terceiro anos do ensino médio foram consideradas elegíveis para a seleção^14^.

As informações foram obtidas por questionário autopreenchido em sala de aula, utilizando um coletor eletrônico de dados modelo LG GM750Q, sob supervisão da equipe do estudo. Além das variáveis sociodemográficas, também foram utilizados os dados referentes ao consumo de bebidas alcoólicas. Essas variáveis foram aferidas por meio das seguintes perguntas:

- Idade em que tomou pelo menos um copo (ou dose) de bebida alcoólica pela primeira vez.
- Dias de ingesta de pelo menos um copo (dose) de álcool, nos últimos 30 dias.
- Copos ou doses consumidas, em média, nos últimos 30 dias.
- Tipo de bebida alcoólica consumida na maioria das vezes.

Medidas de frequência relativa (prevalências) e respectivos intervalos de confiança de 95% foram calculados para o uso de bebidas alcoólicas nos últimos 30 dias, para a frequência desse uso, número de copos ou doses consumidas no período, idade em que bebeu pela primeira vez e tipo de bebida mais consumida. Todos esses dados foram estimados para o País e por macrorregião, sexo e grupo etário.

As estimativas de prevalência e os intervalos de confiança foram corrigidos considerando-se o desenho amostral. As análises foram realizadas usando o comando svy (*survey*) para análise de dados complexos do programa Stata 14.0^b^.

O ERICA foi realizado de acordo com os princípios da Declaração de Helsinki. O estudo foi aprovado pelos Comitês de Ética da Universidade Federal do Rio de Janeiro (Processo 45/2008) e de cada um dos 26 estados e do Distrito Federal. Foram obtidas autorizações para realização do estudo nas secretarias de educação e nas escolas. Todos os alunos assinaram um termo de assentimento. Quando o comitê de ética exigiu o consentimento informado dos pais, mesmo para os alunos que não iriam realizar a coleta de sangue, este foi solicitado ao responsável. A privacidade do aluno e a confidencialidade das informações foram garantidas.

## RESULTADOS

No total, 74.589 adolescentes responderam ao questionário. Desses, 55,3% eram do sexo feminino. A idade média foi de 14,7 anos (DP = 1,6), e 78,7% estudavam em escolas públicas. A Tabela 1 apresenta a distribuição por sexo e idade da amostra e da população estimada após expansão da amostra^14^.

**Tabela 1:** Adolescentes da amostra do ERICA e da população estimada segundo o IBGE, por sexo, grupo etário e macrorregiões em municípios com mais de 100 mil habitantes. ERICA, Brasil, 2013-2014.

Sexo e idade	Brasil	%	Norte	%	Nordeste	%	Centro-Oeste	%	Sudeste	%	Sul	%
Da amostra ERICA												
Masculino	33.364	44,7	6.861	45,5	10.310	44,5	4.097	42,1	7.620	44,6	4.476	46,9
12-14 anos	15.433	20,7	3.167	21,0	4.658	20,1	1.965	20,2	3.587	21,0	2.056	21,6
15-17 anos	17.931	24,0	3.694	24,5	5.652	24,4	2.132	21,9	4.033	23,6	2.420	25,7
Feminino	41.225	55,3	8.212	54,5	12.857	55,5	5.630	57,9	9.460	55,4	5.066	53,1
12-14 anos	18.708	25,1	3.739	24,8	5.853	25,3	2.547	26,2	4.322	25,3	2.247	23,6
15-17 anos	22.517	30,2	4.473	29,7	7.004	30,2	3.083	31,7	5.138	30,1	2.819	29,5

Total	74.589	100	15.073	20,2	23.167	31,1	9.727	13,0	17,080	22,9	9.542	12,8

População estimada												
Masculino	5.095.563	50,2	427.365	4,2	1.082.182	10,7	389.167	3,8	2.595.521	25,6	601.328	5,9
12-14 anos	2.697.440	26,6	224.876	2,2	569.111	5,6	204.588	2,0	1.375.262	13,6	323.603	3,2
15-17 anos	2.398.123	23,6	202.489	2,0	513.071	5,1	184.579	1,8	1.220.259	12,0	277.725	2,7
Feminino	5.052.137	49,8	427.997	4,2	1.082.851	10,7	388.843	3,8	2.557.985	25,2	594.461	5,9
12-14 anos	2.650.761	26,1	225.587	2,2	562.205	5,5	202.923	2,0	1.344.038	13,2	316.008	3,1
15-17 anos	2.401.376	23,7	202.410	2,0	520.646	5,1	185.920	1,8	1.213.947	12,0	278.453	2,7

Total	10.147.700	100	855.362	8,4	2.165.033	21,3	778.010	7,7	5.153.506	50,8	1.195.789	11,8

Fonte da estimativa populacional: http://www.ibge.gov.br/home/estatistica/populacao/projecao_da_populacao/2013/default.shtm

ERICA: Estudos dos Riscos Cardiovasculares em Adolescentes

Vinte e um por cento dos adolescentes consumiram bebidas alcoólicas pelo menos uma vez nos últimos 30 dias, sendo a maior prevalência observada na região Sul (27,5%) e a menor, na região Norte (14,8%). As prevalências aumentaram quando se passou da faixa etária de 12-14 anos para 15-17 anos (Tabela 2).

**Tabela 2:** Prevalências e intervalos de confiança de consumo de bebidas alcoólicas por macrorregião, sexo e grupo etário. ERICA, Brasil, 2013-2014.

Sexo e Idade	Brasil		Norte		Nordeste		Centro-Oeste		Sudeste		Sul	
					
	%	IC95%	%	IC95%	%	IC95%	%	IC95%	%	IC95%	%	IC95%
Masculino	21,0	19,5-22,5	14,7	13,5-16,1	16,8	14,8-19,1	23,2	20,7-26,0	22,1	19,6-24,9	26,3	23,2-29,6
12-14 anos	13,1	11,6-14,8	7,7	6,5-9,1	10,3	8,2-12,9	12,9	10,7-15,6	13,7	11,1-16,9	19,2	16,3-22,6
15-17 anos	29,8	27,7-32,1	22,6	20,5-24,8	24,1	21,6-26,8	34,7	30,2-39,4	31,7	27,9-35,6	34,4	28,9-40,4
Feminino	21,5	20,3-22,7	14,9	13,7-16,2	16,5	15,1-18,1	23,3	21,5-25,1	22,7	20,6-24,9	28,8	25,8-32,1
12-14 anos	14,4	13,1-15,8	10,8	9,4-12,2	10,8	9,3-12,4	14,8	13,3-16,4	15,3	13,1-17,8	19,3	16,1-23,1
15-17 anos	29,3	27,3-31,4	19,5	17,8-21,5	22,8	20,6-25,1	32,5	29,2-36,0	30,9	27,2-34,9	39,6	35,7-43,7

Total	21,2	20,2-22,3	14,8	13,8-15,9	16,7	15,2-18,3	23,3	21,4-25,2	22,4	20,7-24,3	27,5	25,3-29,9

Essas prevalências não foram diferentes quando estratificadas por sexo nas cinco macrorregiões (Tabela 2). Quando os dados foram estratificados por capitais, Porto Alegre, Florianópolis e Vitória apresentaram as maiores prevalências de uso de álcool para ambos os sexos. Natal foi a capital com menor prevalência (Figura 1). No caso dos municípios do interior, as regiões Sul e Centro-Oeste apresentaram as maiores prevalências para ambos os sexos, enquanto as menores prevalências foram observadas nas regiões Norte e Nordeste (Figura 1).

**Figura 1:** Prevalências e intervalos de confiança de consumo de álcool em adolescentes do sexo feminino (A) e masculino (B) por capitais. ERICA, Brasil, 2013-2014.

Dentre os adolescentes que referiram consumo de bebidas alcoólicas nos últimos 30 dias, aproximadamente 68,0% o fizeram em uma ou duas ocasiões no período (Tabela 3). Ao se comparar as faixas de consumo de uma a duas vezes no mês contra 10 ou mais vezes, os adolescentes do sexo masculino e aqueles mais velhos fizeram uso mais frequente de álcool. A região Centro-Oeste foi exceção, sendo maior a proporção de usuários de álcool do sexo masculino no grupo que fez uso uma a duas vezes e do sexo feminino no estrato de seis a nove vezes no último mês.

**Tabela 3:** Frequência (número de dias) de uso de bebidas alcoólicas entre adolescentes que consumiram álcool nos últimos 30 dias, por macrorregião, sexo e grupo etário. ERICA, Brasil, 2013-2014.

Frequência	Brasil		Norte		Nordeste		Centro-Oeste		Sudeste		Sul	
					
	%	IC95%	%	IC95%	%	IC95%	%	IC95%	%	IC95%	%	IC95%
1-2 dias												
Masculino	67,0	64,3-69,5	71,2	66,8-75,3	68,5	63,4-73,2	68,2	63,3-72,8	65,8	61,1-70,1	65,5	60,2-70,4
12-14	73,0	68,7-76,9	78,6	71,4-84,4	74,0	64,4-81,7	78,7	72,3-84,0	71,2	64,1-77,4	71,4	61,4-79,7
15-17	60,2	57,3-63,0	62,9	58,6-67,1	62,4	58,3-66,3	56,6	50,0-63,0	59,7	54,5-64,7	58,6	54,3-62,7
Feminino	69,0	66,2-71,6	76,7	73,9-79,3	73,1	69,4-76,5	61,5	56,6-66,3	67,2	62,0-72,0	68,0	64,5-71,4
12-14	74,1	69,6-78,2	83,5	79,2-87,1	79,1	73,5-83,7	62,2	53,0-70,6	71,2	62,5-78,5	78,7	73,8-82,9
15-17	63,2	60,4-66,0	69,1	64,3-73,5	66,7	62,8-70,5	60,8	56,0-65,4	62,8	57,7-67,7	55,9	50,4-61,3

Total	67,9	65,8-70,0	74,0	71,4-76,3	70,8	67,6-73,8	64,9	60,9-68,7	66,5	62,7-70,1	66,8	63,1-70,3

3-5 dias												
Masculino	16,1	14,5-17,8	12,5	10,1-15,3	11,9	10,2-13,8	14,6	11,2-18,7	18,5	15,8-21,5	17,0	13,2-21,5
12-14	12,3	10,0-15,1	8,3	5,0-13,3	6,2	4,6-8,3	6,9	3,6-12,6	16,2	12,1-21,3	13,1	7,4-22,0
15-17	19,6	17,8-21,5	17,2	14,0-21,0	18,2	15,1-21,6	23,1	17,7-29,6	21,1	17,5-25,2	21,5	18,1-25,4
Feminino	16,6	14,8-18,5	12,3	10,2-14,7	14,5	11,8-17,7	16,3	13,1-20,0	17,8	14,7-21,4	18,1	15,5-21,1
12-14	13,8	11,0-17,3	8,8	6,1-12,6	12,6	8,5-18,4	14,3	9,9-20,2	15,5	10,6-22,1	12,1	8,6-16,7
15-17	19,6	17,8-21,6	16,1	13,2-19,5	16,5	13,6-19,9	18,5	14,7-22,9	20,4	17,3-23,8	25,0	21,0-29,6

Total	16,3	15,0-17,8	12,4	10,7-14,3	13,2	11,6-15,0	15,4	12,7-18,7	18,2	15,8-20,8	17,6	15,4-20,0

6-9 dias												
Masculino	8,4	7,1-9,9	8,4	6,4-10,9	9,1	6,9-11,9	7,1	5,3-9,6	8,0	6,0-10,6	9,6	6,3-14,3
12-14	7,4	5,5-9,9	6,7	4,1-10,7	10,0	6,3-15,5	7,0	4,2-11,4	6,1	3,5-10,5	9,1	4,1-19,0
15-17	9,5	8,0-11,1	10,3	7,6-13,8	8,1	6,2-10,6	7,3	5,4-9,9	10,1	7,6-13,2	10,2	7,7-13,4
Feminino	7,7	6,5-9,1	5,4	3,7-7,7	7,6	5,7-10,0	10,5	7,6-14,4	7,6	5,6-10,3	8,0	6,0-10,6
12-14	5,9	4,3-8,0	3,5	1,7-7,1	3,8	1,8-8,0	11,4	6,6-18,8	6,4	3,9-10,5	5,5	3,2-9,2
15-17	9,6	8,2-11,3	7,4	4,3-12,4	11,6	9,3-14,4	9,5	6,7-13,5	8,9	6,6-12,0	10,8	8,3-14,0

Total	8,0	7,1-9,1	6,9	5,6-8,4	8,3	6,9-10,1	8,8	7,1-11,0	7,8	6,2-9,7	8,8	6,3-12,1

10 ou + dias												
Masculino	8,6	7,3-10,0	7,9	5,3-11,6	10,5	7,8-14,0	10,0	7,5-13,3	7,8	5,8-10,3	8,0	5,8-10,8
12-14	7,3	5,4-9,7	6,4	3,1-12,6	9,8	5,8-16,1	7,5	4,4-12,3	6,5	3,8-10,9	6,4	3,3-12,2
15-17	10,0	8,5-11,8	9,6	7,2-12,7	11,4	7,7-16,5	12,9	9,9-16,7	9,2	7,0-11,9	9,7	6,9-13,5
Feminino	6,8	5,8-8,0	5,7	4,4-7,2	4,8	3,5-6,5	11,7	8,4-16,0	7,4	5,6-9,6	5,8	4,4-7,7
12-14	6,1	4,6-8,1	4,1	2,4-6,9	4,4	2,5-7,9	12,1	7,1-20,1	6,9	4,4-10,5	3,7	1,9-7,0
15-17	7,5	6,3-9,0	7,4	5,7-9,5	5,1	3,9-6,7	11,2	8,3-14,9	7,9	5,7-10,9	8,2	6,1-11,0

Total	7,7	6,8-8,6	6,8	5,2-8,7	7,6	6,2-9,4	10,9	8,4-14,0	7,6	6,2-9,2	6,9	5,6-8,5

Entre os adolescentes que consumiam bebidas alcoólicas, 24,1% beberam pela primeira vez antes de 12 anos de idade, sendo 26,6% (IC95% 22,3-25,9) do sexo masculino e 21,7% (IC95% 19,4-24,2) do feminino. Esse padrão se repetiu em todas as cinco macrorregiões do País.

Os tipos de bebidas alcoólicas mais frequentemente consumidos pelos adolescentes no País foram os drinques à base de vodca, rum ou tequila, seguidos de cerveja (Figura 2). Entretanto, esse padrão variou segundo a macrorregião. No Norte e Nordeste, a cerveja foi a bebida mais referida, enquanto os drinques à base de vodca, rum ou tequila foram os mais referidos nas regiões Centro-Oeste, Sudeste e Sul. De modo geral, houve maior proporção de adolescentes do sexo masculino entre os consumidores de cerveja, e do sexo feminino entre os consumidores de bebidas *Ice* (Tabela 4). O consumo de cerveja e de drinques à base de vodca, rum ou tequila aumentou com a idade, enquanto o de bebidas *Ice* e de vinho diminui.

**Figura 2:** Tipos de bebidas alcoólicas consumidas pelos adolescentes por macrorregião. ERICA, Brasil, 2013-2014.

**Tabela 4:** Percentual dos tipos de bebida consumida pelos adolescentes na maioria das vezes nos últimos 30 dias, por sexo e idade. ERICA, Brasil, 2013-2014.

Características	Cerveja		Vinho		*Ice*		Cachaça		Vodca, tequila e rum		Outros	
					
	%	IC95%	%	IC95%	%	IC95%	%	IC95%	%	IC95%	%	IC95%
Masculino	25,1	22,4-27,9	11,1	9,5-13,0	12,4	10,9-14,2	3,6	2,7-4,7	35,3	32,3-38,5	8,4	7,1-10,0
12-14	23,1	19,3-27,4	13,6	11,0-16,7	13,3	11,4-15,5	3,4	2,1-5,5	30,1	26,3-34,3	10,9	8,5-13,8
15-17	27,3	24,8-29,9	8,3	7,0-10,0	11,5	9,2-14,2	3,7	2,5-5,4	41,1	37,6-44,8	5,7	4,6-6,9
Feminino	19,5	17,7-21,5	12,9	11,0-15,0	19,9	16,9-23,4	2,7	2,0-3,6	33,3	30,4-36,4	10,0	8,5-11,7
12-14	15,5	13,5-17,8	14,4	11,4-18,1	22,8	18,4-27,8	2,2	1,3-3,7	29,5	25,7-33,7	13,4	10,9-16,3
15-17	24,0	21,2-27,0	11,2	9,6-12,9	16,8	13,8-20,3	3,2	2,3-4,5	37,5	33,6-41,5	6,3	5,0-7,8

## DISCUSSÃO

A prevalência de consumo de bebidas alcoólicas em pelo menos uma ocasião nos últimos 30 dias, encontrada no ERICA (21,0%), foi menor do que aquela observada em estudantes brasileiros da Pesquisa Nacional de Saúde do Escolar (PeNSE), realizadas em 2009 (27,3%)^10^ e 2012 (26,1%)^c^. No entanto, a amostra da PeNSE foi restrita aos alunos da nona série, sendo que aproximadamente 90,0% deles tinha idade entre 13 e 15 anos.

Não houve diferença entre os dados de prevalência do ERICA e aqueles encontrados no sexto levantamento com estudantes do ensino fundamental e médio de 27 capitais brasileiras, realizado em 2010^3^.

Pinsky et al.^12^ (2010) relataram prevalência de 24,2% em subamostra de 661 adolescentes brasileiros, com idade entre 14 e 17 anos, que fizeram parte do I Levantamento Nacional de Álcool e Drogas (LENAD), realizado em 2005-2006.

As prevalências de uso de álcool encontradas no Brasil são inferiores àquelas estimadas em estudo nacional conduzido nos EUA em 2013 – *Youth Risk Behavior Survey*, no qual cerca de 35,0% dos adolescentes entre 14 e 17 anos referiram consumo de álcool nos últimos 30 dias^7^. Dados do *Health Behavior in School-Aged Children* (HBSC) *Study*, referentes a adolescentes com 11 a 15 anos de 41 países e regiões da Europa e América do Norte, são mais difíceis de serem comparados com os nossos, pois são apresentados como consumo semanal, variando de 0% a 59,0% dependendo do país, sexo e faixa etária^5^.

Assim como em nosso estudo, os dados sobre adolescentes do LENAD 2005-2006 e da PeNSE 2012 mostraram prevalência mais elevada de uso de álcool em adolescentes residentes na macrorregião Sul do Brasil, e menor para aqueles da região Norte. Entretanto, no caso do LENAD 2005-2006, os autores combinaram as macrorregiões Norte e Centro-Oeste em um mesmo estrato^12^. Um estudo com adolescentes entre 11 e 15 anos da população geral da cidade de Pelotas, na região Sul do País, encontrou prevalência de uso de álcool no último mês de 23,0%^13^. As prevalências de uso de álcool no presente estudo, assim como aquelas relatadas por Pinsky et al.^12^, no LENAD 2005-2006, e por Malta et al.^10^, na PeNSE, aumentaram com a idade, o que sugere a manutenção do consumo por aqueles que iniciaram precocemente, e a adesão de novos usuários com o passar do tempo.

Nossos resultados, quando calculados por macrorregião, são consistentes com os achados de Pinsky et al.^12^ (2010) e de Strauch et al.^13^ (2009), que não observaram diferença na prevalência de usuários de álcool nos últimos 30 dias entre adolescentes dos sexos masculino e feminino. Entretanto, adolescentes do sexo masculino parecem fazer um uso mais intenso, sobretudo se considerarmos a frequência mensal de 10 vezes ou mais.

Um quarto dos adolescentes que referiram consumo de álcool no último mês no presente estudo experimentou pela primeira vez antes dos 12 anos de idade, sendo essa proporção maior no sexo masculino. Outros estudos, nacionais e internacionais, também relatam início precoce na experimentação de bebidas alcoólicas^3^ ^,^ ^7^ ^,^ ^10^ ^,^ ^12^. Esse dado é preocupante se considerarmos que o consumo de bebidas alcoólicas só está legalmente autorizado no Brasil em indivíduos com 18 anos ou mais, e que a iniciação precoce tem sido associada com maior risco de lesões não intencionais, acidentes de carro, comportamento de risco sexual, uso de tabaco e de drogas ilícitas, além de prejuízo cognitivo na vida adulta^9^ ^,^ ^11^.

Quando analisamos os tipos de bebidas mais comumente consumidos, parece que a preferência do tipo de bebida está mudando. Nossos dados foram muito diferentes daqueles relatados por Pinsky et al.^12^, na subamostra de adolescentes do LENAD 2005-2006. No caso deste último, a bebida referida como mais consumida foi a cerveja (cerca de 50,0%), seguida pelo vinho (cerca de 35,0%). De modo geral, nosso estudo encontrou uma referência maior ao consumo de destilados (vodca, rum e tequila), sobretudo nas regiões Centro-Oeste, Sudeste e Sul. Mesmo nas regiões Norte e Nordeste, onde a cerveja foi a bebida mais consumida, o uso de drinques à base de vodca, rum ou tequila ficou em segundo lugar, ultrapassando 20,0% na proporção de bebidas mais consumidas no último mês, independentemente do sexo. Na região Sul, de clima mais frio e produtora de vinhos, este ficou em terceiro lugar entre as bebidas mais consumidas, com cerca de 13,0%. Portanto, observa-se provável mudança do tipo de bebida preferido por adolescentes brasileiros, que estariam optando por bebidas de alto teor alcoólico. Tal hipótese merece ser investigada em estudos futuros com esse grupo populacional, juntamente com as motivações para tal comportamento. Uma explicação a ser considerada seria a busca, pelos adolescentes, do efeito do álcool, mas com um sabor mais atraente, o que é facilmente obtido com a mistura de refrigerantes, sucos e bebidas lácteas. Um estudo com 350 adolescentes e jovens de Sidney, Austrália, identificou os drinques como sendo as bebidas prediletas nessa faixa etária^4^. Outro aspecto a ser ressaltado quanto ao consumo desse tipo de bebida é que, por conter maior concentração alcoólica (em torno de 40,0%), as bebidas destiladas proporcionam os efeitos da alta ingestão de álcool mesmo com consumo de pequenas quantidades.

Os dados do ERICA representam o perfil de consumo de adolescentes que frequentavam escolas e que residiam em municípios de médio ou grande porte, i.e., com mais de 100 mil habitantes. Apesar disso, estes achados se aproximam daqueles referentes aos adolescentes da população geral do Brasil, pois o acesso à escola é de cerca de 95,0% para a população de seis a 14 anos, e de 85,0% na faixa etária de 15 e 19 anos^d^.

Em conclusão, foi observada prevalência elevada de uso de uma substância cujo consumo é proibido por lei na faixa etária coberta por este estudo. Adicionalmente, os dados mostram início precoce do uso de álcool e possível mudança na bebida de preferência em comparação com investigações anteriores. No entanto, os resultados de estudos com populações de estudantes devem ser considerados com cautela quando se pretende generalizar os achados para populações que podem estar fora das escolas. Por esse motivo, estudos com a população geral de adolescentes são necessários para melhor investigar essas tendências.

O conhecimento sobre os padrões de consumo é essencial para se eliminar ideias pré-concebidas no que tange aos grupos mais atingidos e aos padrões de uso de bebidas alcoólicas por adolescentes, fornecendo subsídios para o desenvolvimento de políticas públicas visando a prevenção e o tratamento. De acordo com Laranjeira et al.^8^, a maioria da população adulta no Brasil apoia o aumento de programas preventivos e restritivos ao uso do álcool em escolas e de campanhas governamentais de alerta sobre os seus riscos. Diante disso, são necessárias políticas que regulamentem o mercado do álcool e evitem o consumo precoce de bebidas alcoólicas, sempre acompanhadas de avaliações contínuas da efetividade dessas medidas.

## Footnotes

a Brasil. Lei nº 13.106, de 17 de março de 2015. Altera a Lei nº 8.069, de 13 de julho de 1990 - Estatuto da Criança e do Adolescente, para tornar crime vender, fornecer, servir, ministrar ou entregar bebida alcoólica a criança ou a adolescente; e revoga o inciso I do art. 63 do Decreto-Lei nº 3.688, de 3 de outubro de 1941 - Lei das Contravenções Penais. Brasília (DF); 2015 [citado 2015 nov 12]. Disponível em: http://www.planalto.gov.br/ccivil_03/_Ato2015-2018/2015/Lei/L13106.htm

b Stata Corp. Stata: statistical software for professionals. Version 14.0. College Station, Texas; 2015.

c Instituto Brasileiro de Geografia e Estatística. Pesquisa Nacional de Saúde do Escolar: 2012. Rio de Janeiro (RJ): IBGE; 2013.

d Instituto Brasileiro de Geografia e Estatística. Pesquisa Nacional de Saúde do Escolar: 2012. Rio de Janeiro (RJ): IBGE; 2013.
